# The Bundibugyo Ebola Virus Emergency and the erosion of global health security

**DOI:** 10.1371/journal.pgph.0006648

**Published:** 2026-07-08

**Authors:** Boghuma K. Titanji

**Affiliations:** Division of Infectious Diseases, Emory University School of Medicine, Atlanta, Georgia, United States of America; PLOS: Public Library of Science, UNITED STATES OF AMERICA

The World Health Organization has officially declared an outbreak of Ebola in northeastern Democratic Republic of Congo (DRC) (It uri Province), the 17th in the nation's history [1]. It is caused by a very rare species of Ebola virus, the Infundibuli ebolavirus species, which until now had been responsible for only two recorded outbreaks in humans. The first occurred in Uganda in 2007, leading to the virus's discovery [2]; the second followed a few years later in the DRC.

In an unprecedented move, the WHO declared the outbreak a Public Health Emergency of International Concern (PHEIC) before convening its Emergency Committee, the first time a Director-General has done so [3], signaling the seriousness of the situation. What caught the international community's attention was the scale of the outbreak; as of mid-May 2026, 600 suspected cases and 139 deaths had been reported [1], making it the largest outbreak ever recorded of this rare Bundibugyo ebola species. Several factors make this a significant regional threat; a rare virus for which we have no readily deployable vaccines or therapeutics; an epicenter in a part of the DRC beset by ongoing conflict that has driven large-scale population displacement; and porous borders shared with several East African countries, including South Sudan, Rwanda and Uganda. Two cases, one of them fatal, have already been confirmed in neighboring Uganda, both in the capital, Kampala [3].

Both the DRC and Uganda have extensive experience responding to outbreaks of deadly hemorrhagic viruses; indeed, the last Ebola outbreak in the DRC ended only in December 2025. What has changed dramatically is the landscape of global public health funding under the current US political leadership alongside continued tensions and damaging rhetoric directed at multilateral organizations such as the WHO. The dismantling of USAID has left a large unfilled gap, compromising the regional structures crucial for disease surveillance and outbreak response [4,5]. What has emerged to fill the void are bilateral memoranda of understanding between the US and African governments that tie health aid to mineral deals and to the one-way transfer of health and pathogen data. Some of the proposed agreements have been roundly condemned as exploitative and reminiscent of colonial extraction [6,7].

In addition, the US has withdrawn from the WHO [4], resulting in a significant loss of funding to the organization (it was its single largest country donor), as well as the loss of vital technical expertise and support [4]. The US decision has since been copied by Argentina, which completed its own withdrawal in March 2026 [8]. The WHO's leadership had warned that these moves would weaken the organization and its ability to coordinate responses that depend on multinational collaboration. It has not taken long for that warning to be tested. A Hantavirus (Andes virus) outbreak aboard a cruise ship carrying nationals of 23 countries demanded intense coordination and close cooperation between governments to keep it from growing into something far larger [9].

None of this is to dismiss the legitimate concerns behind these decisions. Many have argued that the WHO was overdue for reform, that no single nation should carry so large a share of its budget, and that decades of donor-driven aid have too often entrenched dependency instead of building self-reliant health systems [4]. Advocates of the new bilateral model present it as a route to genuine partnership and country ownership, and many African governments rightly aspire to finance, and one day manufacture, their own countermeasures rather than wait on the goodwill of others. These are fair points, and the old arrangement was far from perfect. But reforming a system is not the same as dismantling it in the middle of an emergency, and partnership is not a contract signed under duress that trades a population's health data for access to its minerals. Self-reliance built by withdrawing support before any alternative exists is not sovereignty; it simply transfers the risk onto those least able to absorb it.

**Fig 1:**
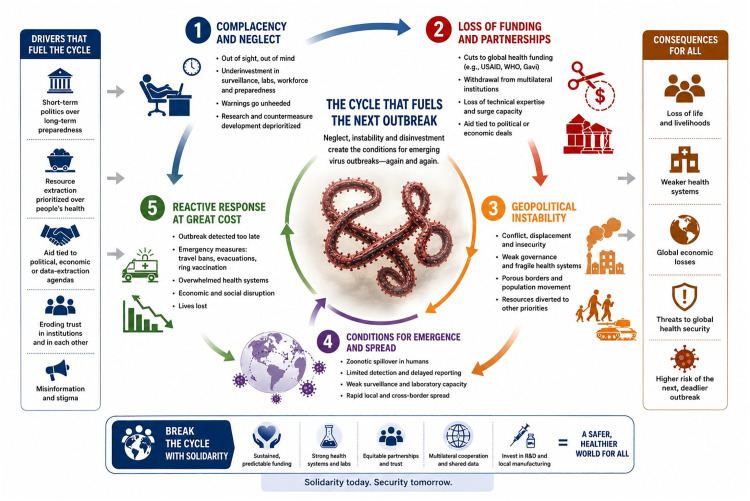
The cycle fueling the next outbreak. Conceptual model illustrating how neglect, funding disruptions, weakening of multilateral partnerships, geopolitical instability, and delayed responses interact to increase vulnerability to emerging infectious disease outbreaks. Figure conceptualized by the author and created with AI-assisted (Chatgpt) illustration tools, with author refinement.

As we watch the events unfold in the DRC, it is increasingly evident that a global community of 8 billion people cannot keep itself safe without solidarity and international collaboration. The recurring cycle of neglect followed by reactive, after-the-fact response continues to reveal itself as a model that will never succeed (Fig 1). Bundibugyo virus was discovered nearly two decades ago, in 2007; it demonstrated then that it could cause large and serious outbreaks, yet we were still caught unprepared. The US holds a patent [2] covering the virus isolated during the 2007 outbreak, tied to research and development of vaccines and therapeutics, yet no vaccine for this virus exists today, nor was its development ever prioritized. The existence of a patent is not the same as a development pathway - it is evidence that scientific capacity existed, while the political and commercial incentives to move a product forward did not. The parallels with the Ebola epidemic in West Africa are glaring. It took the Zaire virus killing more than 11,300 people in Sierra Leone, Liberia and Guinea [10], for vaccine candidates that had languished for years to finally move into human trials, ultimately yielding the two licensed Ebola Zaire vaccines we have today [11,12].

In its response to the Bundibugyo outbreak, the US has once again led with travel restrictions, the only country to do so, barring entry to all non-citizens who have been in the DRC, South Sudan or Uganda within the previous 30 days [13]. Measures calibrated to the incubation period are a defensible instinct, but the issue is not caution itself, but who is granted its protection. A US citizen who contracted the virus [14] has been airlifted to Germany for treatment much as nationals of Europe and North America were evacuated to safety while Ebola tore through three West African countries in 2014–2016. The frontline workers in Bunia, Ituri, and the surrounding communities at the epicenter of this outbreak do not have the same privilege yet they are working under extraordinarily difficult conditions to keep us all safe.

History will keep repeating itself unless we resolve to change it. The regional gains made in pandemic preparedness across Africa since the West African Ebola epidemic are why we can now move from outbreak identification to a shared virus sequence in days; the reason recent outbreaks of Sudan virus and Marburg in Tanzania, Rwanda and Uganda have been rapidly contained; and the reason we now have ready-to-deploy Ebola Zaire vaccines, targeted monoclonal antibodies, and rapid diagnostic tests for field use when outbreaks occur.

Retreating on global public health preparedness efforts through funding disruptions and the withdrawal of support for key agencies such as the WHO, Gavi and others is profoundly short-sighted and will reverse the significant gains that have been made. Ignoring the regional conflicts sustained by misplaced priorities that value mineral extraction above the lives of vulnerable communities only helps create the perfect storm for the next outbreak. The question is not whether there will be another outbreak, or whether there are deadlier viruses still undiscovered, waiting to exploit the fault lines we help create. The question is whether the world's leadership will finally choose to see the basic humanity of vulnerable communities and act to protect them before it is too late. This is not just the right thing to do; it is the only appropriate human response.
